# Receptor Specificity and Transmission of H2N2 Subtype Viruses Isolated from the Pandemic of 1957

**DOI:** 10.1371/journal.pone.0011158

**Published:** 2010-06-21

**Authors:** Claudia Pappas, Karthik Viswanathan, Aarthi Chandrasekaran, Rahul Raman, Jacqueline M. Katz, Ram Sasisekharan, Terrence M. Tumpey

**Affiliations:** 1 Influenza Division, Centers for Disease Control and Prevention, Atlanta, Georgia, United States of America; 2 Harvard-Massachusetts Institute of Technology Division of Health Sciences and Technology, Koch Institute of Integrative Cancer Research, Department of Biological Engineering, Massachusetts Institute of Technology, Cambridge, Massachusetts, United States of America; Tsinghua University, China

## Abstract

Influenza viruses of the H2N2 subtype have not circulated among humans in over 40 years. The occasional isolation of avian H2 strains from swine and avian species coupled with waning population immunity to H2 hemagglutinin (HA) warrants investigation of this subtype due to its pandemic potential. In this study we examined the transmissibility of representative human H2N2 viruses, A/Albany/6/58 (Alb/58) and A/El Salvador/2/57 (ElSalv/57), isolated during the 1957/58 pandemic, in the ferret model. The receptor binding properties of these H2N2 viruses was analyzed using dose-dependent direct glycan array-binding assays. Alb/58 virus, which contains the 226L/228S amino acid combination in the HA and displayed dual binding to both alpha 2,6 and alpha 2,3 glycan receptors, transmitted efficiently to naïve ferrets by respiratory droplets. Inefficient transmission was observed with ElSalv/57 virus, which contains the 226Q/228G amino acid combination and preferentially binds alpha 2,3 over alpha 2,6 glycan receptors. However, a unique transmission event with the ElSalv/57 virus occurred which produced a 226L/228G H2N2 natural variant virus that displayed an increase in binding specificity to alpha 2,6 glycan receptors and enhanced respiratory droplet transmissibility. Our studies provide a correlation between binding affinity to glycan receptors with terminal alpha 2,6-linked sialic acid and the efficiency of respiratory droplet transmission for pandemic H2N2 influenza viruses.

## Introduction

Among the several known hemagglutinin (HA) and neuramindase (NA) subtypes of influenza A viruses [1], only three subtypes (H1N1, H2N2 and H3N2) have been documented to cause widespread and sustained disease in humans. While H1N1 and H3N2 subtypes continue to circulate in humans, the H2N2 Asian pandemic virus of 1957/58 (responsible for approximately 2 million deaths globally), disappeared from the human population after 1968 [2]. The 1957 pandemic H2N2 strain originated as a reassortant in which a novel avian-like HA replaced the prevailing human adapted H1 HA surface protein [3]. Genetic analysis of the 1957 strain and circulating avian H2 strains indicated that the pandemic virus also acquired a novel polymerase subunit PB1 gene and NA gene from an influenza A virus of wild waterfowl origin [4].

While much of the concerns of an influenza pandemic have been due to exceptionally virulent strains of the H5N1 avian influenza virus, there is also concern about the possibility of pandemic virus recycling in humans [5], [6]. More recently, this concern has been heightened by the emergence of a novel H1N1 strain whose global spread and mortality rate prompted the World Health Organization (WHO) to declare the first pandemic of the 21^st^ century [7]. Like the current pandemic H1N1 virus, certain strains of H2 are swine-origin reassortant viruses containing genes derived from avian and swine influenza viruses and appear to be more adapted to mammals [8], [9]. Due to the occurrence of several genetic changes prior to displacement in the human population around 1968, individuals born after that year lack specific B cell immunity to the H2 HA [10]. Moreover, surveillance of influenza viruses in avian species has provided evidence that H2 viruses are isolated on a yearly basis and some avian H2N2 viruses, circulating in wild and domestic birds, are antigenically similar to the 1957 pandemic strain [11], [12].

One of the characteristics of a pandemic influenza A virus is its ability to transmit efficiently via respiratory droplets between humans. We and others have made substantial effort to develop a system using ferrets as a model to investigate both direct contact and respiratory droplet transmission of influenza A viruses [13], [14], [15], [16]. We have further established a correlation between transmissibility in ferrets and biochemical binding specificity of the HA protein to sialylated glycan receptors for H1N1 viruses [13], [17], [18]. The human adaptation of influenza A virus is associated with the ability of the HA to change its binding preference from glycan receptors present in the avian gastrointestinal tract that consist of terminal α2,3-linked SA (referred to as α2,3) [19] to those present in the human upper respiratory tract that possess terminal α2,6-linked SA (referred to as α2,6) [20], [21], [22]. In addition to adaptation of the HA, the adaptation of the polymerase basic 2 (PB2) protein was recently found to be necessary for influenza virus transmission in experimentally inoculated mammals. In particular, the amino acids 627 and/or 701 of PB2 have been related to not only transmissibility but also to replication efficiency of viruses [15], [23].

Most of the above studies have focused on the H1N1, H3N2 and H5N1 subtypes, and very little on H2N2 viruses. The glycan receptor binding preference of H2N2 viruses has been characterized using solid phase binding assays [24] and more recently using crystal structures [25], [26]. These studies point to the importance of amino acid 226 in defining the binding specificity of HA to human α2,6 or avian α2,3 glycan receptors. However, the transmissibility of these viruses in the ferret model as it relates to glycan-receptor binding preference has not been established. An understanding of the mechanisms behind receptor binding preference and how this property influences efficient virus transmission of pandemic strains can aid in the surveillance and response to future outbreaks of H2N2 viruses in the human population.

In this study, we used the ferret model that recapitulates the efficient transmission of seasonal influenza viruses and the poor transmission of avian influenza viruses in humans [13], [14], [15], [16]. The glycan receptor binding preference of the H2N2 viruses was characterized using agglutination of red blood cells (RBCs) followed by a more extensive dose-dependent direct glycan array binding assay. An avian H2N2 virus (A/Mallard/New York/6750/78; Mallard/78) failed to spread efficiently to naïve ferrets, whereas the human H2N2 Alb/58 virus spread efficiently by respiratory droplet transmission. Although a 1957 isolate, ElSalv/57, failed to spread efficiently to naïve ferrets, strikingly a natural variant was isolated from one of the contact ferrets in a rare transmission event and showed improved transmission to naïve ferrets compared to the wild type virus. Characterization of the H2N2 variant revealed a key mutation (glutamine to leucine at position 226) in the HA that was sufficient to change its receptor preference from α2,3 to α2,6 SA.

## Results

We selected two human H2N2 viruses, ElSalv/57 and Alb/58 that were isolated during the Asian pandemic of 1957/58. ElSalv/57 virus was isolated from the Southern hemisphere in the first year of the pandemic whereas Alb/58 virus was isolated in 1958 from the Northern hemisphere. We also selected Mallard/78, which is among the first avian H2N2 viruses isolated in the late 1970s as a representative avian virus for the transmission studies.

### Transmissibility of avian Mallard/78 virus

**Figure 1:**
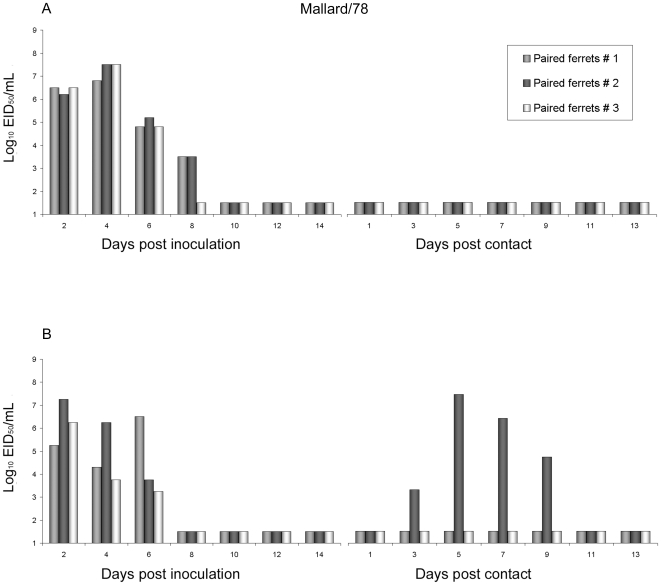
Respiratory droplet and direct contact transmission experiments using avian H2N2 virus. For each experiment, three ferrets were inoculated intranasally (i.n.) with 10^6^ EID_50_ of Mallard/78 virus and placed in individual cages. **A**: Twenty four hours later, one naïve ferret was housed in a cage adjacent to each of the cages containing an inoculated ferret (respiratory droplet transmission), separated by perforated walls to allow the exchange of respiratory droplets. **B**: For direct transmission experiments, one naïve ferret was housed in the same cage with the inoculated ferret. Nasal wash samples were collected from the inoculated and contact ferrets on alternating days and titered in eggs. Bars with the same color represent an inoculated/contact ferret pair. The limit of virus detection was 10^1.5^ EID_50_/ml.

The ferret model was used to evaluate the pathogenesis and transmissibility of selected H2N2 influenza viruses. For respiratory droplet transmission experiments, three animals were inoculated i.n. with 10^6^ plaque forming units (PFU) of virus. Approximately 24 hours later, inoculated-contact animal pairs were established by placing a naïve ferret in each of three adjacent cages with perforated side walls, allowing exchange of respiratory droplets in the absence of direct or indirect contact [16]. Transmission was assessed by titration of infectious virus in nasal washes and detection of virus specific antibodies in convalescent serum. Ferrets inoculated with the avian Mallard/78 virus did not cause any visible clinical symptoms or significant weight loss and failed to transmit by respiratory droplets, despite efficient replication in the ferret upper respiratory tract of inoculated animals (Figure 1A). Virus titers in the nasal washes of inoculated ferrets achieved peak mean titers of 10^7.3^ EID_50_/ml on day four post inoculation (p.i.) (Table 1). Sneezing was not observed at any time point among the three inoculated ferrets. These results indicate that, like avian influenza viruses of other subtypes, the avian H2N2 virus failed to transmit efficiently between ferrets by means of respiratory droplets.

**Table 1:** Clinical signs, virus replication, seroconversion, and transmission in ferrets inoculated with H2N2 viruses.

		*Number of inoculated ferrets/total number*					*Number of contact ferrets/total number*	
		Clinical Signs			Mean nasal wash titer log_10_EID_50_/ml (peak day)	Seroconversion (HI range)**	Virus detected in nasal wash	Seroconversion (HI range)**
Virus	Receptor binding specificity	Mean Maximum Weight Loss (%)*	Sneezing (day(s) of onset)	Lethargy (day of onset)				
**Mallard/78 (RD)** π	α2,3/2,6	3.5	0/3	0/0	7.3 (4)	3/3 (80–160)	0/3	0/3
**Mallard/78 (DC)** ¶		4.8	1/3 (4)	0/0	6.25 (2)	3/3 (80–160)	1/3	1/3 (320)
**Alb/58 (RD)**	α2,6/2,3	7	3/3 (4–6)	0/0	6.2 (2)	3/3 (320–640)	3/3	3/3 (320–640)
**Alb/58 (DC)**		9	2/3 (3–7)	2/3 (3)	5.9 (2)	2/2 (320)	3/3	3/3 (320)
**ElSalv/57 (RD)**	α2,3	7.2	1/3 (4)	1/3 (4)	6.5 (2)	3/3 (320)	1/3	1/3 (160)
**ElSalv/57 (DC)**		4.5	0/0	2/3	7.0 (2)	3/3 (320)	0/3	1/3 (640)
**El Salv/57-Q226L (RD)**	α2,6	2.6	1/3 (7)	1/3 (7)	6.25 (2)	3/3 (320–640)	3/3	2/3 (160–640)***

RD, respiratory droplet; DC, direct contact transmission; ND, not determined.

π This experiment was performed using virus that was obtained from plaque purification and grown in MDCK cells.

¶ These experiments were done independently from the first one, using virus that was grown in allantoic fluid of 10-day old embryonated eggs.

*The percentage corresponds to the ferrets that exhibited highest drop in weight during the experiment.

** Hemagglutination inhibition (HI) titer is shown. Performed with homologous virus and turkey red blood cells.

*** The lack of seroconversion in one of the ferrets is explained by the short window span between late peak of virus detection and sera sample collection.

Mallard/78 virus was examined further for its ability to transmit under conditions where ferrets were housed together in the same cage (direct contact transmission). Virus titers in the nasal washes of inoculated ferrets achieved peak mean titers of 10^6.25^ EID_50_/ml on day 2 p.i. (Table 1, Figure 1B). Transmission of Mallard/78 virus was inefficient as virus was detected in the nasal washes of only one of three contact ferrets and transmission was confirmed by seroconversion to the inoculating strain (Table 1). Sneezing was present in only one (on a single day) out of three Mallard/78 virus-inoculated ferrets and the paired ferret for that particular cage was positive for Mallard/78 virus on day 4 post-contact (p.c.).

### Transmissibility of human H2N2 viruses

**Figure 2:**
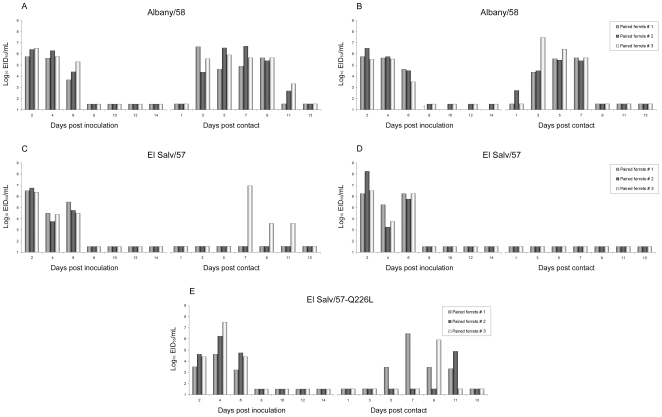
Respiratory droplet and direct contact transmission experiments using human H2N2 viruses. For each experiment, three ferrets were inoculated i.n. with 10^6^ EID_50_ of Albany/58, El Salv/57 or El Salv/57-Q226L mutant. Each ferret was placed in individual cages. **A, C and E**: Twenty four hours later, one naïve ferret was housed in a cage adjacent to each of the cages containing an inoculated ferret (respiratory droplet transmission), separated by perforated walls to allow the exchange of respiratory droplets. **B and D**: For direct transmission experiments, one naïve ferret was housed in the same cage with the inoculated ferret. Nasal wash samples were collected from the inoculated and contact ferrets on alternating days and titered by EID_50_. Bars with the same color represent an inoculated/contact ferret pair. The limit of virus detection was 10^1.5^ EID_50_/ml. †, ferret found dead on day 8 p.i.

Ferrets inoculated with Alb/58 virus generally displayed 7–9% mean maximum weight loss and some lethargy at the height of virus infection. Five out of six ferrets recovered fully from inoculation; however, one Alb/58-inoculated ferret died on day 8 p.i. after exhibiting respiratory signs (sneezing and nasal discharge) and 13% weight loss shortly before death (Table 1, Figure 2A and B). Alb/58 virus achieved peak mean nasal wash titers of 10^6.2^ to 10^5.9^ EID_50_/ml and had sustained titers of ≥10^4.0^ EID_50_/ml for 6 days p.i. Sneezing was observed among the majority of Alb/58 inoculated ferrets and the virus transmitted efficiently by respiratory droplet as well as by direct contact transmission (Figures 2A and 2B). Virus was detected in the nasal washes of the contact ferrets between 1 to 3 days p.c. and all animals seroconverted to Alb/58 virus (Table 1). These results show that similar to other human influenza viruses, the human-adapted H2N2 Alb/58 virus was capable of efficient transmission to naïve ferrets by both direct contact and respiratory droplets.

Ferrets inoculated with ElSalv/57 virus exhibited mild lethargy and weight loss similar to Alb/58-inoculated ferrets. Sneezing was present in only one of six ElSalv/57-inoculated ferrets (sneezing in one animal on day 4 p.i.) (Table 1). ElSalv/57 virus reached similar peak titers as the Alb/58 virus in nasal washes (peak titers, EID_50_/ml = 10^6.5–7.0^), however ElSalv/57 virus did not transmit efficiently by respiratory droplets and failed to transmit by direct contact as evidenced by the paucity of virus shedding and seroconversion among the contact ferrets (Figures 2C and D, Table 1). Remarkably, virus was detected in the nasal washes of one of the respiratory droplet contact ferrets late in the course of the experiment (Figure 2C). Seroconversion occurred in this contact ferret (ferret # 3) as well as in all three inoculated ferrets (Table 1). This isolated virus transmission event led us to investigate whether a natural mutant virus had arisen during the course of this experiment.

### Characterization and transmissibility of the H2N2 natural variant virus

The unique transmission event of an ElSalv/57 virus in one ferret prompted further characterization of the virus that was shed on day 7 p.c. It should be noted that this transmission event occurred unusually late compared to viruses that transmit efficiently in this model [15], [16]. RNA was extracted from virus present in the nasal wash collected from the contact ferret and was subjected to RT-PCR and sequence analysis. Sequence analysis identified a natural variant (designated ElSalv/57-Q226L) possessing a single amino acid change in the glycan-receptor binding site; glutamine (Q) to leucine (L) at position 226. Amino acid position 226 of the H2 HA represents a key residue that influences binding to SA [20], [21], [26], [27]. Only two other amino acid changes occurred in the ElSalv/57-Q226L virus HA, one on the HA1 portion of the molecule at position 16; a change from alanine (A) to valine (V) (H3 numbering), and a second change located on the HA2 at position 460, a change from aspartic acid (D) to asparagine (N). Both changes map to regions of unassigned functions within the HA protein.

The HA of ten to twelve plaque-purified viruses derived from the nasal washes of all three ElSalv/57 inoculated ferrets were sequenced. Although a heterogeneous distribution of viruses with different receptor binding specificities was detected, no virus displaying the variant L226/G228 genotype was isolated from the virus-donor inoculated ferret (Ferret #3) (Table 2; receptor binding specificity characterized using RBC agglutination (Figure S1 and Table S1). Instead, a combination of Q226/G228 and Q226/S228 was present in a 1∶1 proportion in the nasal washes collected on days four and six p.i. (Table 2). Virus from nasal wash collected earlier in infection (day 2 p.i.), revealed that all three ElSalv/57-inoculated ferrets had the same Q226/G228 amino acid combination as the ElSalv/57 wild type virus, suggesting that a G228S mutation occurred in ferret #3 after inoculation. Screening of plaque purified viruses from the two remaining inoculated ferrets (days four and six p.i.) that did not transmit virus revealed that their amino acid combination was either identical to ElSalv/57 wild type virus (Ferret #1), or a combination of Q226/G228 and Q226/S228 in a proportion of 1∶9 (Ferret #2) (Table 2).

**Table 2:** Composition of amino acids responsible for receptor binding among plaque purified viruses obtained from ferret nasal washes.

	Inoculated Ferrets			Contact Ferrets		
	Position 226 (codon) Number/total**	Position 228 (codon) Number/total**	Receptor Binding Specificity	Position 226 (codon) Number/total	Position 228 (codon) Number/total	Receptor Binding Specificity
**Input virus (used for inoculation)**	Q (CAA) 10/10	G (GGT) 10/10	α 2,3	-	-	-
**Ferret #1**	Q (CAA) 10/10	G (GGT) 10/10	α 2,3	ND*	ND*	-
**Ferret # 2**	Q (CAA) 10/10	G (GGT) 1/10 S (AGT) 9/10	α 2,3 none	ND*	ND*	-
**Ferret #3**	Q (CAA) 12/12¶	G (GGT) 6/12 S (AGT) 6/12	α 2,3 none	L (CTA) 10/10	G (GGT) 10/10	α 2,6

* No detectable ElSalv/57 virus was isolated from nasal washes (day 6 p.i.) during a respiratory droplet transmission experiment.

** Refers to the number of plaque purified viruses containing that specific codon in relation to the number of numbers of plaque purified viruses that were analyzed by nucleotide sequencing.

¶ Same results were obtained in the nasal wash samples collected on day 4 p.i.

The plaque-purified ElSalv/57-Q226L virus was then assessed for its ability to transmit by respiratory droplets. Similar to previous transmission experiments, three ferrets were inoculated with10^6^ EID_50_/ml of ElSalv/57-Q226L virus. ElSalv/57-Q226L-infected ferrets exhibited peak mean nasal wash titers on day 2 p.i. (EID_50_/ml = 10^6.25^), similar to titers achieved in ferrets inoculated with the wild type ElSalv/57 virus (Table 1). However, in contrast to wild type ElSalv/57 virus, the ElSalv/57-Q226L virus transmitted efficiently to all naïve ferrets (Figure 2E). These results suggest that single amino acid changes in the receptor binding site of the H2 HA may be sufficient to confer transmission via respiratory droplets. To understand the differences in transmissibility with relation to the structural and biochemical properties of the HA, we next characterized the glycan receptor binding preference of the avian and human (wild-type and natural HA variants) H2 viruses using RBC agglutination and glycan array methodologies.

### Glycan-receptor binding specificity of H2 viruses from 1957/58 pandemic

**Figure 3:**
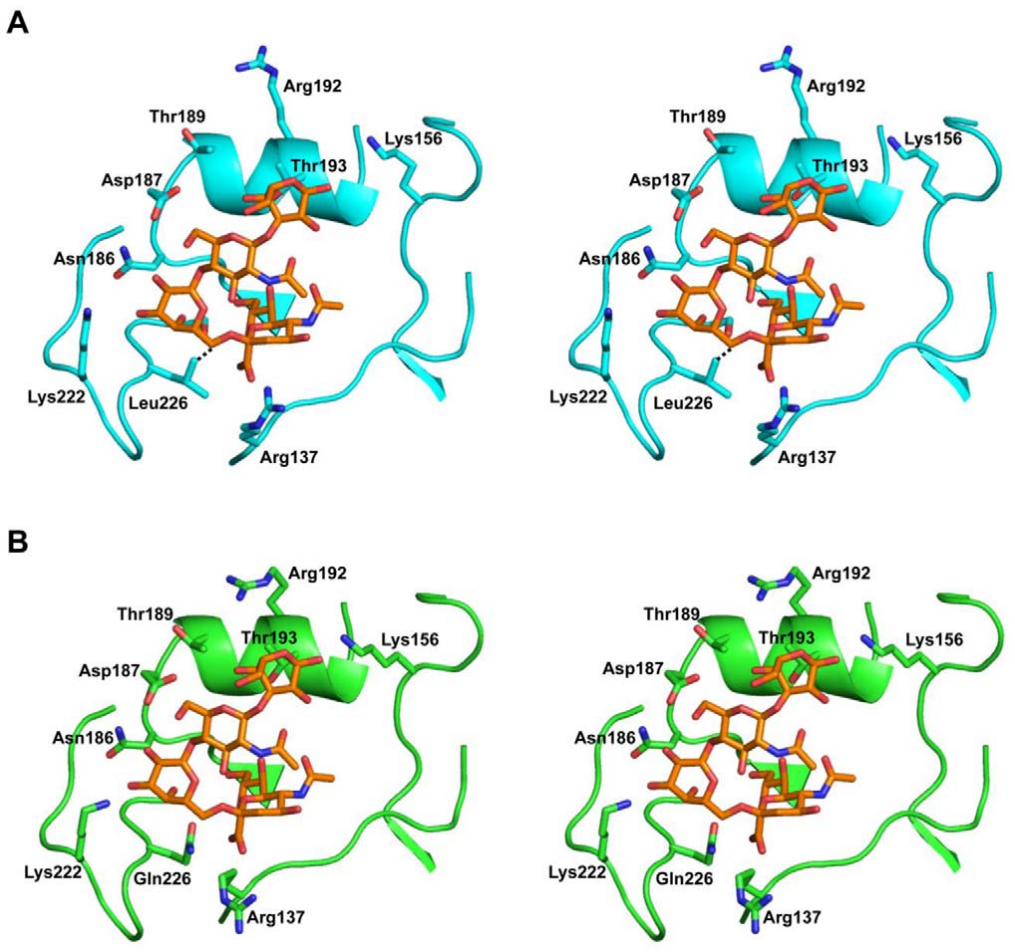
Structural model of human H2N2 viruses with α2,6 receptor. **A**, Stereo view of homology based structural model of Alb/58 HA complexed with α2,6 oligosaccharide. This model was constructed using a representative human H2N2 HA – α2,6 receptor co-crystal structure (PDB ID: 2WR7) as template. The optimal contact between the critical Leu226 with the C6 atom of SA α2,6-linked to Gal motif is shown with dashed line. **B**, Stereo view of homology based structural model of ElSalv/57 HA complexed with α2,6 oligosaccharide. This model was constructed using an avian-like H2N2 HA (PDB ID: 3KU3) as template. The main differences in contacts between A and B are in positions 226 and 228 that make contacts with SA α2,6-linked to the penultimate Gal and in 156 position that makes contacts with additional sugars at the reducing end of penultimate Gal. The sugar is shown as a stick representation colored by atom (carbon in *orange*, oxygen in *red* and nitrogen in *blue*). The homology based structural models were constructed as described previously [18].

The recently solved X-ray co-crystal structures of avian and human H2 HAs with representative α2,3 and α2,6 SA liked glycan receptors [26] provides insights into the key amino acids involved in optimal contacts with these receptors (Figure 3). In addition to the conserved residues that bind to the SA – Ser/Thr136, Trp153, Thr155, His183, Glu190, Leu194 (numbering is based on H3 HA) – the key residue positions that are involved in making contacts with the other sugars of the glycan receptors are shown in Table 3. Among these positions (numbered based on H3 HA), amino acids at 137, 222, 226, 228 are involved in making contacts with the SA and the penultimate Gal sugar in both α2,3 and α2,6 glycan receptors. On the other hand, amino acids at 156, 189, 192 and 193 are either directly or indirectly involved in making contacts with additional sugars at the reducing end the penultimate Gal of an α2,6 SA linked tetra-pentasaccharide (for example multiple lactosamine repeats terminated by α2,6 SA) (Figure 3).

**Table 3:** Composition of the hemagglutinin receptor binding amino acids and residues that interact with α2-3 and α2-6 glycans.

	Amino Acid Position (H3 numbering)							
	137	156	189	192	193	222	226	228
**Consensus Human H2N2 1962–1968**	K	K	A	R	A	K/E*	L	S
**Consensus Human H2N2 1957–1961**	R	K	T	R	T	K	L/Q**	S/G**
**Consensus Avian H2N2**	R	K	A/T*	R	T	K	Q	G
**Alb/58**	R	K	T	R	T	K	L	S
**ElSalv/57**	R	K	T	R	T	K	Q	G
**Mallard/78**	R	K	T	R	T	K	Q	G

Consensus sequences from human H2N2 viruses that circulated during pandemic, H2N2 avian viruses and from the viruses used in this study.

*More than one amino acid is shown in cases where the proportion of viruses containing it was higher than 30%.

**Receptor binding amino acids 226Q228G and 226Q228S could be found in the viruses that circulated during this period.

Examination of the HA sequences of the three H2N2 viruses used in this study reveal differences in the glycan receptor-binding site. The primary differences in the H2N2 viruses are at positions 226 and 228 of HA. While the Alb/58 virus possesses L226 and S228, both ElSalv/57 and the avian H2N2 Mallard/78 virus have Q226 and G228 in these positions (Table 3). L226 and S228 make optimal contacts with SA α2,6-linked to the Gal of a human glycan receptor [26]. Among these two residues L226 plays a crucial role in preferentially binding to the α2,6-linked as compared to α2,3-linked SA. Based on these amino acid differences in the glycan receptor binding site of the H2 HAs, Alb/58 virus was expected to have a predominant binding preference to α2,6 as compared to α2,3 glycan receptors. On the other hand, both ElSalv/57 and Mallard/78 were likely to preferentially bind α2,3 over α2,6 glycan receptors. The hypothesized binding properties were tested experimentally.

A hemagglutination assay with H2 viruses binding to turkey red blood cells (RBCs) resialylated with either α2-3 or α2-6 linked sialosides was used as a preliminary screen of receptor specificity (Figure S1, Table S1). Alb/58 and Mallard/78 viruses were found to agglutinate both α2,6 and α2,3 sialylated RBCs, in contrast to the binding properties observed in a previous study that used a solid-phase receptor binding assay [24]. The differential hemagglutination titers achieved suggested that Alb/58 virus possessed stronger binding preference for α2,6 SA in comparison to α2,3 SA receptors, whereas Mallard/78 virus appeared to possess comparable preference for both receptors in this assay (Table S1). However, ElSalv/57 virus preferentially agglutinated α2,3 sialylated RBCs over α2,6 sialylated RBCs, consistent with previous studies [24]. The natural variant (Q226L HA mutant) of ElSalv/57 virus showed preferential agglutination of α2,6 sialylated RBCs as compared to α2,3 sialylated RBCs.

Previously we demonstrated that high relative binding affinity of HA to α2,6 glycan receptors with a characteristic structural topology is an important determinant for human adaptation of influenza A viruses [17]. Given the observed differences in the transmissibility between the human H2N2 viruses (including the natural variant), we analyzed in detail the direct binding of these viruses in a dose-dependent fashion to a glycan array comprised of structurally well-defined α2,3 and α2,6 oligosaccharides. For this analysis, five glycans were used that differ in their linkage, length and characteristic structural topology as described previously [17].

**Figure 4:**
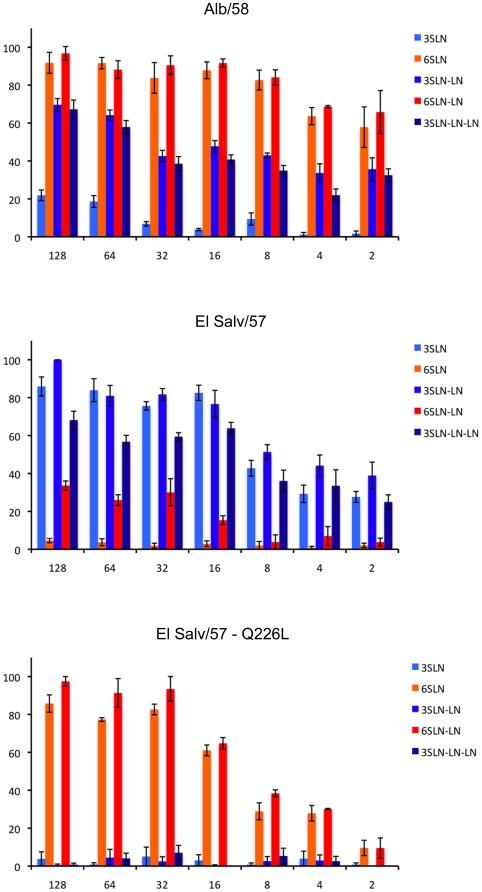
Glycan binding of Alb/58, ElSalv/57, and A/El Salv/57-Q226L viruses. The binding signals are expressed as percentage of maximum binding of each virus to the glycan arrays. At saturating HA titers, both Alb/58 and A/El Salv/57-Q226L showed binding signals to long and short α2-6 glycans, but Alb/58 also showed high binding signals to long and short α2-3 glycans. ElSalv/57 virus showed binding affinity to all three types of α2-3 glycans but also displayed some binding to long α2-6 glycans at higher titers. The types of glycans used on the arrays are described (Table S2).

Alb/58 virus showed binding signals for both α2,6 as well as α2,3 glycans (Figure 4, Table S2). The substantial binding of a human influenza virus to glycans with α2-3 SA is notable since this pattern is generally not characteristic of human-adapted influenza H1 and H3 subtype viruses, including the 1918 (SC/18:H1N1) and 1968 (H3N2) pandemic strains [13], [17], [24]. However, the significantly higher binding signals for α2,6 glycans (6′SLN and 6′SLN-LN) as compared to α2,3 glycans over the entire range of viral titers demonstrated the high relative binding affinity of Alb/58 virus to α2,6 glycans. On the other hand, ElSalv/57 virus showed preferential binding to α2,3 oligosaccharides over α2,6 oligosacharides (Figure 4). The binding of the ElSalv/57 natural variant (carrying the Q226L HA mutation) showed predominant binding signals to α2,6 and only minimal binding signals to α2,3 glycan receptors (Figure 4). Comparison of the α2,6 binding signals over the entire viral titer range between Alb/58 and the ElSalv/57 natural variant viruses shows that Alb/58 virus has a higher α2,6 binding affinity than the natural variant.

The above experimental binding data support the predicted binding properties of the H2N2 viruses based on structural and sequence analysis of the glycan receptor binding sites on the HA. Furthermore, the α2,6 binding affinity of ElSalv/57 and Alb/58 human H2N2 viruses correlates with their observed transmissibility via respiratory droplets to naïve ferrets. The improved respiratory droplet transmissibility of the natural variant of ElSalv/57 virus correlates with the dramatic increase in its binding specificity to α2,6 glycan receptors.

## Discussion

The factors leading to the emergence of a pandemic influenza virus are complex and poorly understood. An understanding of the molecular and biologic requirements for efficient transmissibility is critical for the early identification of a potential pandemic virus and the application of optimal control measures. Despite the fact that an H2N2 virus was responsible for a major pandemic in the 20^th^ century, the absence of this subtype in the human population in the last 40 years has resulted in the H2 subtype receiving little attention. Moreover, the proportion of humans that lack serologic immunity to the H2 subtype is ever expanding. Thus, if a contemporary avian H2 virus were to jump the species barrier and acquire the ability to undergo efficient and sustained transmission among humans, the conditions could be set for an influenza epidemic or even pandemic. Our study is the first to characterize the transmission of H2N2 pandemic viruses in mammals and reestablish the relationship between transmissibility and glycan-receptor binding properties of these viruses.

The efficient transmission of Alb/58 virus was consistent with ferret transmission results obtained for other known human influenza viruses, including the 1918 (SC/18:H1N1) pandemic virus [13], [15]. In contrast, ElSalv/57, another H2N2 virus isolated from human host did not transmit efficiently. However, isolation and characterization of the natural variant (ElSalv/57-Q226L) isolated from the nasal wash of the contact ferret revealed a receptor binding site (Q226L) mutation in the HA. The amino acids at positions 226 and 228 have been implicated to play an important role in distinguishing α2,3 and α2,6 glycan receptors [24]. Furthermore, when this natural variant obtained from nasal wash of the contact ferret was plaque purified and further tested this virus demonstrated substantially improved transmission via respiratory droplet as compared to the parental ElSalv/57 virus.

In the ferret model, efficient respiratory droplet transmission of avian influenza viruses with a α2,3 SA receptor binding preference has not been observed [15], [16]. Furthermore, mutations in HA that enable a switch from the human α2,6 to the avian α2,3 SA receptor binding preference result in a virus incapable of respiratory droplet transmission [13]. Characterization of the glycan-binding preference of the H2N2 viruses showed that the avian H2N2 virus, Mallard/78, and the ElSalv/57 virus preferentially bound to α2,3 glycan receptors. The dual binding of Mallard/78 to both types of receptors could be explained by recent results that showed the ability of avian H2 HA to bind to both human and avian receptors (Figure 3 and [26]). The inefficient transmission of these viruses is consistent with the previously established relationship between α2,3 glycan-receptor binding preference and poor transmission of avian-adapted H1, H5, H7 and H9 viruses [14], [15], [16], [28]. The Alb/58 virus showed dual binding to both α2,6 and α2,3 glycan receptors with its α2,6-binding affinity substantially higher than its α2,3-binding affinity (as observed in dose-dependent direct glycan array binding assay). This binding pattern is different from the one obtained with the 1918 (SC/18:H1N1) pandemic strain, which binds exclusively to a specific subset of α2,6 sialylated oligosaccharides (including 6′SLN-LN) [17]. Nevertheless, the efficient transmission of Alb/58 virus was consistent with ferret transmission results of human influenza viruses, including the 1918 (SC/18:H1N1) pandemic virus [13], [29]. While some mixed binders such as the 1918 HA variant A/New York/1/1918 or A/Hong Kong/213/03 (H5N1) virus do not transmit efficiently [13], [30], other mixed binders like A/Texas/36/91 (H1N1) virus do spread efficiently [13] thereby deomonstrating that mixed binding does not necessarily disqualify a virus from transmission. Analysis of the glycan-binding specificity of the natural variant of the ElSalv/57 virus that carries the Q226L mutation in the HA showed a dramatic shift in the glycan binding specificity to α2,6 glycan receptors in comparison with its parental virus. These results contrast with the recent results of Xu et al. [25], which observed that H2 HA 226L/228G produce weak binding to both α2,6 and α2,3 linked glycans. In the current study, the increase α2,6 glycan-binding specificity of ElSalv/57 virus correlated with the increased respiratory droplet transmission to naïve ferrets.

Based on the transmission data obtained on multiple avian and human-adapted viruses over the past several years it is improbable that an influenza virus possessing a strong α2,3 SA receptor preference would spread efficiently among humans [13], [16]. Nevertheless, the question still remains of whether H2N2 viruses with α2,3 SA receptor binding specificity were circulating early in the pandemic [20], [24], [31]. Assessing this possibility includes providing an understanding of whether and how these early-circulating viruses could switch receptor binding specificity. Such studies may provide additional information on the mechanisms of how influenza could adapt to a new host. Notably, Alb/58, a human-adapted and high passage virus, seemingly possesses α2,3 glycan-binding, in addition to α2,6 SA binding; this double binding feature has previously been noted for H2N2 viruses [20]. Although a number of H2N2 pandemic viruses possessed α2,3 SA receptor preference, internal genes from these viruses (including the natural variant ElSalv/57-Q226L identified here) possess characteristics of human-adapted viruses. In particular, the H2N2 pandemic virus possessed a human adapted PB2 gene. Adaptation of the PB2 also appears to be critical for efficient aerosolized respiratory transmission of influenza virus [15], [23]. Specifically, a single amino acid substitution from glutamic acid to lysine at amino acid position 627 supports efficient influenza virus replication at the lower temperature (33°C) found in the mammalian airway, and contributes to efficient transmission in ferrets and guinea pigs.

In our study, many of the plaque-purified viruses obtained from the three inoculated ferrets nasal washes collected later in the course of infection did not show a homogeneous population at amino acids 226 and 228. Thus, the different residues within receptor binding site of the H2 HA appear to confer a selective advantage by enabling a receptor specificity switch allowing for transmission of a variant virus. Given these results, the receptor binding site of the H2 HA appears to be genetically unstable allowing the virus to switch its binding preference during replication. Finally, because the avian and human-adapted H2N2 viruses that were isolated early in the pandemic share similarities in their glycan-binding amino acids (Table 3), we hypothesize based on our results that these amino acids provide a molecular framework that allows the easy switch of their receptor binding preferences. Together with changes in PB2, these HA changes allow for efficient transmission of virus in a human host.

## Materials and Methods

### Viruses

H2N2 viruses were provided by the Viral Surveillance and Diagnostic Branch (VSDB), CDC. Original human H2N2 virus isolates or low-egg-passage isolates are not available for study. A/Mallard/New York/6750/78 (Mallard/78) was previously passaged seven times in embryonating hen's eggs and two times in chicken kidney cells; the virus stock used for the experiments was plaque purified and grown in Madin-Darby canine kidney (MDCK) cells [32]. A/Albany/6/58 (Alb/58) virus was passaged nine times in hen's eggs; the virus stock was grown in the allantoic cavity of 10–11 day old hen's eggs. GenBank accession numbers for Mallard/78 and Alb/58 viruses are available from http://www.ncbi.nlm.nih.gov/genomes/FLU/FLU.html. A/El Salvador/2/57 (El Salv/57; GenBank accession no. submitted) virus had been previously passaged eight times in eggs and virus stock was grown in MDCK cells. El Salv/57-Q226L virus (GenBank accession no. submitted) was obtained by plaque purification from the nasal wash of a contact ferret during a transmission experiment and grown in MDCK cells. The EID_50_ titer for each stock was calculated by the method of Reed and Muench following serial titration in eggs [33]. The PFU titer for each cell-grown stock was also determined by standard plaque assay [34]. Viruses were confirmed by RT-PCR and sequencing. Comparisons between parental ElSalv/57 and El Salv/57-Q226L viruses' entire genomes revealed two amino acid changes in the HA (A24V in HA1 and D135N in HA2), two changes in the neuraminidase (V20A and F22L) and three changes in the matrix protein (R57K, G105R and S224R). Research with H2 viruses was conducted under biosafety level 3 containment, including enhancements required by the U.S. Department of Agriculture/CDC Select Agent Program.

### Ethics Statement

All animal research described in this study was specifically approved by CDC's Institutional Animal care and Use Committee (IACUC). The animal research was conducted under the guidance of CDC's IACUC and in an Association for Assessment and Accreditation of Laboratory Animal Care International-accredited facility.

### Ferret Transmission Experiments

Male Fitch ferrets 6–12 months of age (Triple F Farms, Sayre, PA) were used in transmission studies. Ferrets were housed throughout each experiment in cages within a Duo-Flo Bioclean mobile clean room (Lab Products, Seaford, DE) and were determined to be negative for antibody to circulating influenza A (H1N1, H3N2) and Influenza B viruses. Ferrets were inoculated with 10^6^ PFU or EID_50_ of virus in a total volume of 1 ml (500 µL/nostril) as described [32]. Respiratory droplet and contact transmission experiments were conducted as previously described [16]. Briefly, 24 hours after inoculation, uninfected ferrets were housed in either adjacent transmission cages (respiratory droplet transmission) or in the same cage (direct contact transmission). Temperature, weight and clinical symptoms were recorded daily for 14 days. During this period, nasal washes were collected every two days for determination of viral titers in both inoculated and contact ferrets. Convalescent serum from all ferrets was collected days 18–21 p.i./p.c. and tested for H2 HA antibodies by using homologous virus and 0.5% turkey or 1% horse red blood cells as described [35].

### Hemagglutination Assays

Hemagglutination assays using resialyated turkey red blood cells were performed as previously described [34], [36] with minor modifications. Turkey red blood cells were enzymatically desialyated followed by resialylation using either α2-6-(N)-sialyltransferase (Japan Tobacco Inc, Iwata, Japan) or α2-3-(N)-sialyltransferase (Calbiochem, San Diego, CA). Assays were performed by using both 4 and 8 hemagglutination units of virus yielding identical results.

### Dose dependant direct binding assay

Virus stocks were propagated in the allantoic cavity of 10-d-old embryonated hens' eggs at 37°C. The allantoic fluids were harvested 24 h after inoculation and inactivated by treatment with Beta-propiolactone (BPL; 1/1,000) for 3 days at 4°C. Virus binding to the glycan-coated wells was performed as described previously [17] by adding appropriate amount of virus to each well after diluting in PBS containing 1% BSA and incubating overnight at 4°C. After rinsing excess virus with PBS containing 0.05% Tween-20 and PBS, the wells were incubated with antibody against the virus (ferret-anti-influenza A raised against El Salv/57 and Alb/58) for 5h at 4°C. After extensive washing of the antibody, the plate was incubated with HRP-linked goat-anti-ferret antibody (Rockland Immunochemicals) for 2 h at 4°C. After extensive washes with PBS containing 0.05% Tween-20 and PBS, in all cases, HRP activity was estimated using Amplex Red Peroxidase Assay Kit (Invitrogen) according to manufacturer's instructions. Appropriate negative controls were included.

## Supporting Information

Figure S1 Receptor binding specificities using RBC agglutination. Hemagglutination assay using turkey red blood cells (tRBCs) resialylated with either α2,3 or α2,6 sialyl transferases. A/Texas/36/1991 and A/Duck/NY/15024/1996 were used as human and avian controls, respectively. Turkey red blood cells were enzymatically desialyated followed by resialylation using either α2-6-(N)-sialyltransferase (Japan Tobacco Inc, Iwata, Japan) or α2-3-(N)-sialyltransferase (Calbiochem, San Diego, CA). Assays were performed by using 8 hemagglutination units of virus.
(2.04 MB DOC)

Table S1 Composition of the receptor binding amino acids present in the hemagglutinin of H2N2 viruses used in this study and their receptor binding specificity. The viruses were assayed by hemagglutination using resialylated turkey red blood cells (RBCs).
(0.04 MB DOC)

Table S2 Glycans used on arrays for detecting long and short α2-3/α2-6 sialylated chains. Key: Neu5Ac: N-acetyl D-neuraminic acid; Gal: D-galatose; GlcNAc: N-acetyl D-glucosamine. α/β: anomeric configuration of the pyranose sugars. All the sugars are linked via a spacer to biotin (-Sp-LC-LC-Biotin as described in http://www.functionalglycomics.org/static/consortium/resources/resourcecored5.shtml).
(0.07 MB DOC)
