# Supplementary material for: Receptor Specificity and Transmission of H2N2 Subtype Viruses Isolated from the Pandemic of 1957
Source: PLoS One. 2010 Jun 21;5(6):e11158. doi: 10.1371/journal.pone.0011158 (PMC2888575; doi:10.1371/journal.pone.0011158)
Supplement: Figure S1 — Receptor binding specificities using RBC agglutination. Hemagglutination assay using turkey red blood cells (tRBCs) resialylated with either α2,3 or α2,6 sialyl transferases. A/Texas/36/1991 and A/Duck/NY/15024/1996 were used as human and avian controls, respectively. Turkey red blood cells were enzymatically desialyated followed by resialylation using either α2-6-(N)-sialyltransferase (Japan Tobacco Inc, Iwata, Japan) or α2-3-(N)-sialyltransferase (Calbiochem, San Diego, CA). Assays were performed by using 8 hemagglutination units of virus. (2.04 MB DOC) [file pone.0011158.s001.doc]

**Figure S1. Hemagglutination assay of influenza viruses using differentially sialylated turkey RBCs.**

**tRBCs desial. α 2-3 α 2-6**

**tRBC tRBC tRBC**


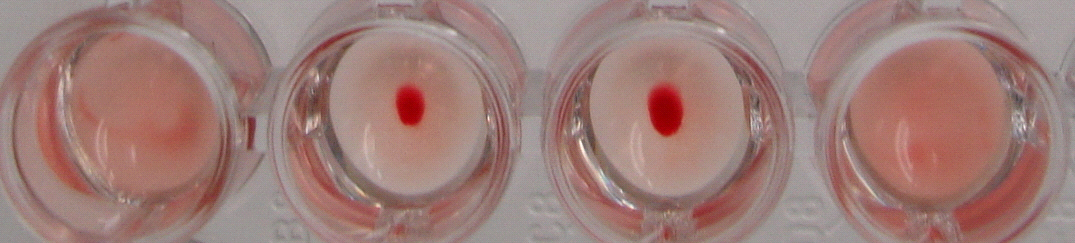

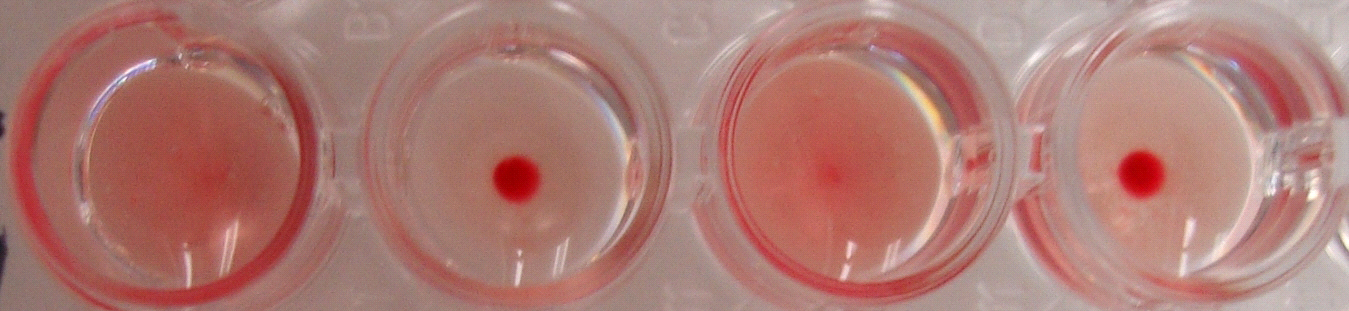

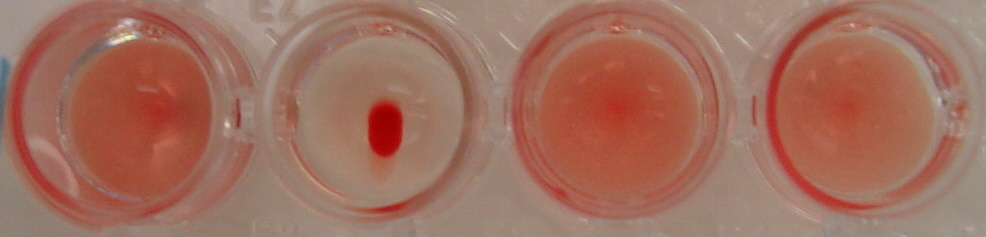

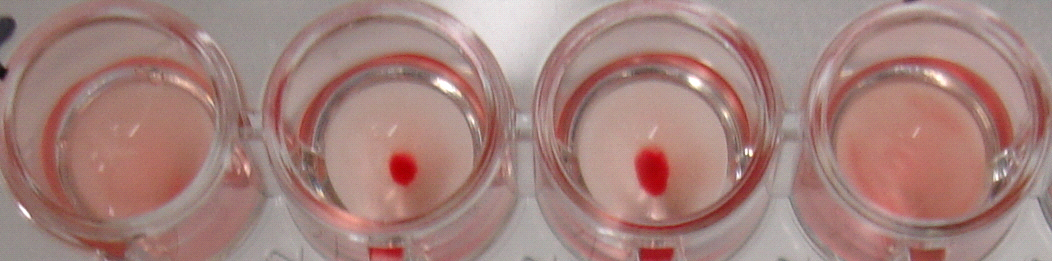

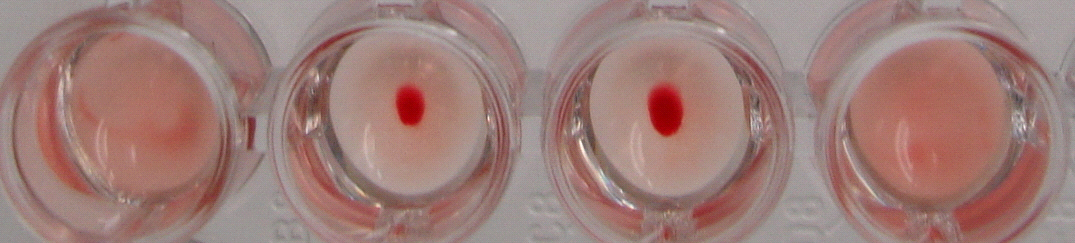

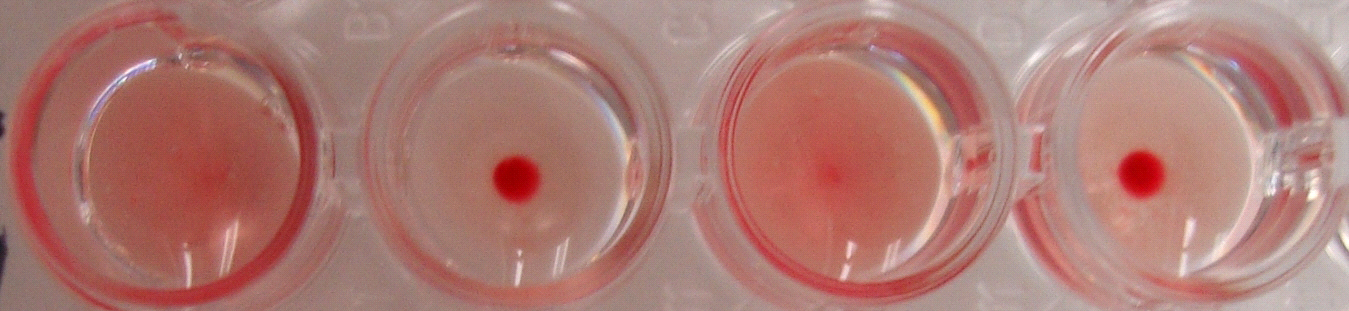

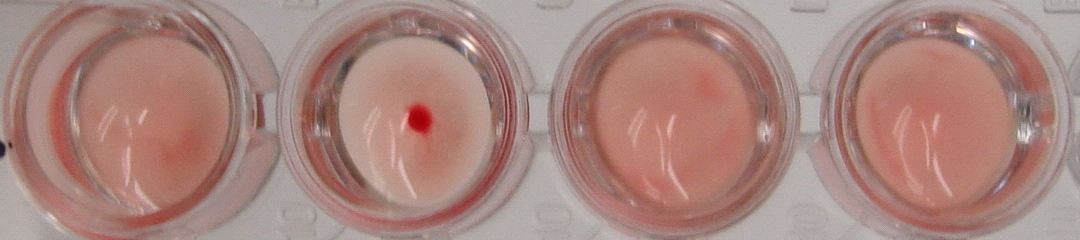

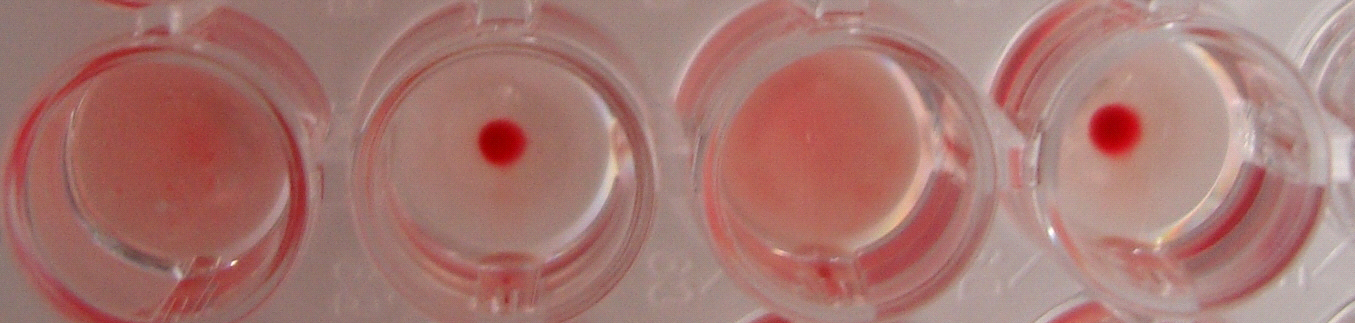


**Human H1N1**

**Avian H1N1**

**Avian H2N2**

**(Mallard/78)**

**Human H2N2**

**(Alb/58)**

**Human H2N2**

**(ElSalv/57-Q226L**

**Human H2N2**

**(Alb/58)**

**Human H2N2**

**(ElSalv/57-Q226L**

**Human H2N2**

**(ElSalv/57)**

**Human H2N2**

**(ElSalv/57-Q226L)**
