# Supplementary material for: Receptor Specificity and Transmission of H2N2 Subtype Viruses Isolated from the Pandemic of 1957
Source: PLoS One. 2010 Jun 21;5(6):e11158. doi: 10.1371/journal.pone.0011158 (PMC2888575; doi:10.1371/journal.pone.0011158)
Supplement: Table S1 — Composition of the receptor binding amino acids present in the hemagglutinin of H2N2 viruses used in this study and their receptor binding specificity. The viruses were assayed by hemagglutination using resialylated turkey red blood cells (RBCs). (0.04 MB DOC) [file pone.0011158.s002.doc]

**Table S1.**  Composition of the receptor binding amino acids present in the hemagglutinin of H2N2 viruses used in this study and their receptor binding specificity.

|  | **Receptor binding amino acids** | |  |  | **Hemagglutination titers of tRBCs re-sialylated with α 2,3 or α2,6 sialic acids** | | |
| --- | --- | --- | --- | --- | --- | --- | --- |
| **Virus** | **Position 226 (codon)** | **Position 228 (codon)** | **Receptor Binding Specificity** |  | **tRBC** | **α 2,3 tRBC** | **α 2,6 tRBC** |
| **Mallard/78** | Q (CAA) | G (GGG) | α 2,3  α 2,6 |  | 256 | 256 | 256 |
| **Alb/58** | L (CTA) | S (AGT) | α 2,6  α 2,3 |  | 64 | 64 | 256 |
| **ElSalv/57** | Q (CAA) | G (GGT) | α 2,3 |  | 128 | 512 | No binding |
| **ElSalv/57-LG** | L (CTA) | G (GGT) | α 2,6 |  | 256 | No binding | 256 |
