# Supplementary material for: Receptor Specificity and Transmission of H2N2 Subtype Viruses Isolated from the Pandemic of 1957
Source: PLoS One. 2010 Jun 21;5(6):e11158. doi: 10.1371/journal.pone.0011158 (PMC2888575; doi:10.1371/journal.pone.0011158)
Supplement: Table S2 — Glycans used on arrays for detecting long and short α2-3/α2-6 sialylated chains. Key: Neu5Ac: N-acetyl D-neuraminic acid; Gal: D-galatose; GlcNAc: N-acetyl D-glucosamine. α/β: anomeric configuration of the pyranose sugars. All the sugars are linked via a spacer to biotin (-Sp-LC-LC-Biotin as described in http://www.functionalglycomics.org/static/consortium/resources/resourcecored5.shtml). (0.07 MB DOC) [file pone.0011158.s003.doc]

**Table S2.**  Glycans used on arrays for detecting long and short α2-3/α2-6 sialylated chains

| **Glycan** | **Expanded IUPAC nomenclature** |
| --- | --- |
| 3’SLN | Neu5Acα2-3Galβ1-4GlcNAcβ1- |
| 6’SLN | Neu5Acα2-6Galβ1-4GlcNAcβ1- |
| 3’SLN-LN | Neu5Acα2-3Galβ1-4GlcNAcβ1-3Galβ1-4GlcNAcβ1- |
| 6’SLN-LN | Neu5Acα2-6Galβ1-4GlcNAcβ1-3Galβ1-4GlcNAcβ1- |
| 3’SLN-LN-LN | Neu5Acα2-3Galβ1-4GlcNAcβ1-3Galβ1-4GlcNAcβ1-3Galβ1-4GlcNAcβ1- |
